# Clinical Benefits of Golden-*Antrodia Camphorata* Containing Antroquinonol in Liver Protection and Liver Fat Reduction After Alcoholic Hepatitis

**DOI:** 10.3389/fphar.2022.757494

**Published:** 2022-06-21

**Authors:** Yu-Ting Yen, Joo-Hyun Park, Seung-Hyun Kang, Today Su, Howard Cheng, Wu-Che Wen, Shin-Shiou Lin, Yu-Ling Tai, Pei-Ni Chen, Shih-Chang Tsai

**Affiliations:** ^1^ Drug Development Center, Institute of New Drug Development, Institute of Biomedical Sciences, China Medical University, Taichung, Taiwan; ^2^ Department of Family Medicine, Korea University Ansan Hospital, Korea University College of Medicine, Ansan, South Korea; ^3^ Clinical Research Center of H PLUS Yangji Hospital, Seoul, South Korea; ^4^ Golden Biotechnology Corporation, New Taipei City, Taiwan; ^5^ Department of Biological Science and Technology, China Medical University, Taichung, Taiwan

**Keywords:** antroquinonol, *Antrodia camphorata*, liver function, aspartate aminotransferase/alanine aminotransferase ratio, anti-lipid function

## Abstract

**Objective:** It has been reported that antroquinonol extracted from Golden-*Antrodia camphorate* exerts protective effects on liver function both *in vitro and in vivo*. However, the protective effects of Golden-*Antrodia camphorata* on liver function have not been fully investigated in human clinical studies. Therefore, the present study aimed to evaluate the beneficial effects of Golden-*Antrodia camphorata* on hepatic function after alcohol consumption in human subjects.

**Methods:** A total of 80 participants with increased γ-glutamyl transferase levels (60–180 U/L) were enrolled in the current study and were randomly divided into two groups. Participants in the first group were orally administrated with 300 mg/day Golden-*Antrodia camphorata* (tablets), while those in the second group received placebo tablets for 12 weeks. Biochemical routine blood tests were performed at 6 and 12 weeks following the first administration.

**Results:** At 12 weeks post the first Golden-*Antrodia camphorata* administration, the serum levels of aspartate aminotransferase (AST; *p* < 0.0001), alanine aminotransferase (ALT; *p* = 0.0002) and triglyceride (*p* = 0.0158) were notably declined in the Golden-*Antrodia camphorata* treatment group compared with the placebo group. No clinically significant differences were observed between the Golden-*Antrodia camphorata* treatment and placebo groups in terms of general safety parameters.

**Conclusion:** A statistically significant difference was obtained in the serum levels of AST, ALT and triglycerides between the Golden-*Antrodia camphorata* and placebo groups. However, no clinical significance was observed in any of the safety parameters examined. Overall, these findings indicated that treatment with Golden-*Antrodia camphorata* exerted protective effects on liver function.

## Graphical Abstract

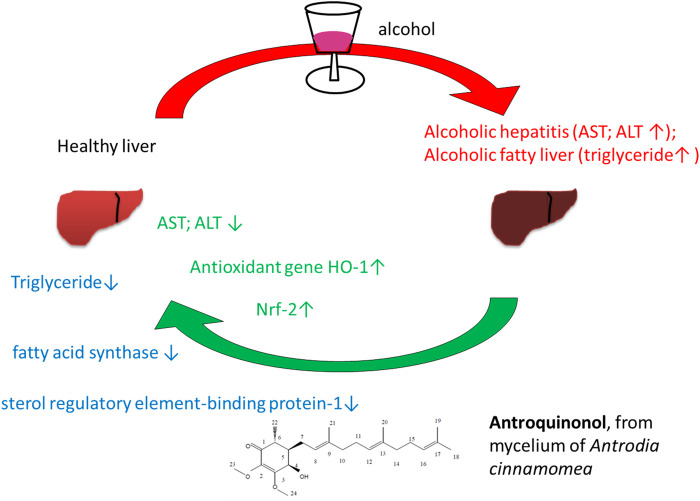

## Introduction

Alcoholic hepatitis most commonly occurs in individuals with mild or chronic alcohol consumption history and may lead to liver damage, which has a high short-term mortality rate of ∼40% within 1 month of clinical presentation (Lucey et al., 2009; Singal et al., 2014; Thursz et al., 2015) demonstrated that treatment of patients with severe alcoholic hepatitis with prednisolone or pentoxifylline resulted in a 90-days mortality rate of ∼30%. Additionally, it has been reported that long-term alcohol abuse increases the risk of developing liver diseases (Younossi and Guyatt, 1998). Gutierrez-Ruiz *et al* showed that heavy alcohol intake could promote the production of several factors, including cytokines such as TNF-α, aspartate aminotransferase (AST), alanine aminotransferase (ALT) and reactive oxygen species (ROS), thus resulting in the pathogenesis and progression of alcohol-induced liver diseases (Gutierrez-Ruiz et al., 1999).

Oxidative stress, produced during alcohol metabolism, may cause liver cell injury. Liver cell injury-induced alcoholic liver diseases account for ∼20% of all alcohol-related liver diseases (Lucey et al., 2009; Serste et al., 2018). This can occur as four by-products of alcohol metabolism can mediate liver cell injury, which in turn may lead to steatohepatitis, gangrene, liver cirrhosis and/or hepatocellular carcinoma. Alcohol consumption may also cause the overproduction of free radicals, which may result in the spontaneous depletion of hepatic glutathione (Papanicolas et al., 2018). However, the overproduction of free radicals not only causes liver damage, but may also lead to vascular injury and lipid peroxidation in the blood, which in turn can promote the development of several diseases. Those who abuse alcohol long-term may develop alcoholic fatty liver disease (AFLD), which may lead to liver cancer (Osna et al., 2017). Antrodia camphorata is a well-known Chinese medicine that suppresses the generation of ROS and *in vitro* studies have suggested that its antioxidative properties are beneficial in reducing liver cell injury (Kumar et al., 2011; Yi et al., 2020).

Antrodia *camphorata*, also known as “niu-chang-chih”, is a precious and common medicinal fungus in Taiwan. Therefore, it is claimed as a “national treasure of Taiwan” (Liu et al., 2012). Taiwanese aborigines alleviate the discomfort caused by excessive alcohol consumption by chewing raw fruits or drinking fruit decoction to minimize alcohol hangover (Ao et al., 2009). Currently, the fruiting bodies of Antrodia *camphorata* have been widely used to treat liver diseases, hypertension, allergies and cancer (Ao et al., 2009). Geethangili *et al* suggested that Antrodia *camphorata*and its bioactive compounds exhibited numerous bioactive properties, including anticancer, hepatoprotective and neuroprotective functions (Ao et al., 2009; Geethangili and Tzeng, 2011). Antrodia *camphorata* contains several bioactive compounds such as flavonoids, terpinoids, polyphenolics and polysaccharides (Chiang et al., 2010). Antroquinonol, a major active compound of Golden-*Antrodia camphorata*, displays anticancer and anti-inflammatory properties (Chiang et al., 2010; Geethangili and Tzeng, 2011). Antirroquinonol from *Antrodia camphorata* protects against ethanol-induced oxidative stress in hepatic cell lines by activating Nrf-2 and HO-1 (Kumar et al., 2011).

Golden-*Antrodia camphorata* has not been studied thoroughly in human clinical studies for its safety and protective effect on liver function. In this study, we sought to assess the beneficial effects of Golden-*Antrodia camphorata* on human hepatic function after alcohol consumption. *Antrodia camphorata* containing antroquinonol has been found to have clinical and pharmacological benefits in numerous cancerous studies, including breast, lung, pancreatic, colon, brain, and liver cancer (Angamuthu et al., 2019; Ganesan et al., 2019). In addition, antroquinonol has been approved by the US FDA for clinical trials (ClinicalTrials.gov Identifier: NCT02047344). Lu *et al* demonstrated that mycelia from Antrodia *camphorata* exerted protective effects against ethanol-induced liver damage in rats (Lu et al., 2007). However, the effects of Golden-Antrodia *camphorata* on lipid metabolism and protection of liver function have not been evaluated in human clinical studies. The results of the present study revealed that antroquinonol extracted from the ethanolic extracts of mycelium of Golden-Antrodia *camphorata* exhibited hepatoprotective effects.

## Materials and Methods

### Participants

This study was registered at the Clinical Research Information Service (CRIS; registration no. KCT0003692; research unique no. 2018AS0229; https://cris.nih.go.kr/cris/search/detailSearch.do/15180). In addition, the study protocol was approved by the Institutional Review Board of the Korea University Ansan Hospital (approval no. 2018AS0229) and H Plus Yangji Hospital (approval no. HYJ 2018-04–006-006) prior to the initiation of the study. The entire study was performed in accordance with the Declaration of Helsinki and the ethical standards provided by the Korean Good Clinical Practice guidelines. The current multicenter randomized, double blind, placebo-controlled study was conducted between 26 December 2018 and 4 November 2020.

All subjects were recruited from the Clinic Trial Center for Functional Foods at Korea University Ansan Hospital and H Plus Yangji Hospital. Written informed consent was obtained from all participants prior to enrollment in this study. The inclusion criteria were as follows: (1) Men or women, aged 20–75 years (range, 21-72) at the time of the screening test; (2) subjects with serum γ-glutamyltranspeptidase (γ-GTP) levels of 60–180 U/L; and (3) subjects who fully understood the detailed description of the study and voluntarily agreed to participate.

### Treatment Regimen

**FIGURE 1:**
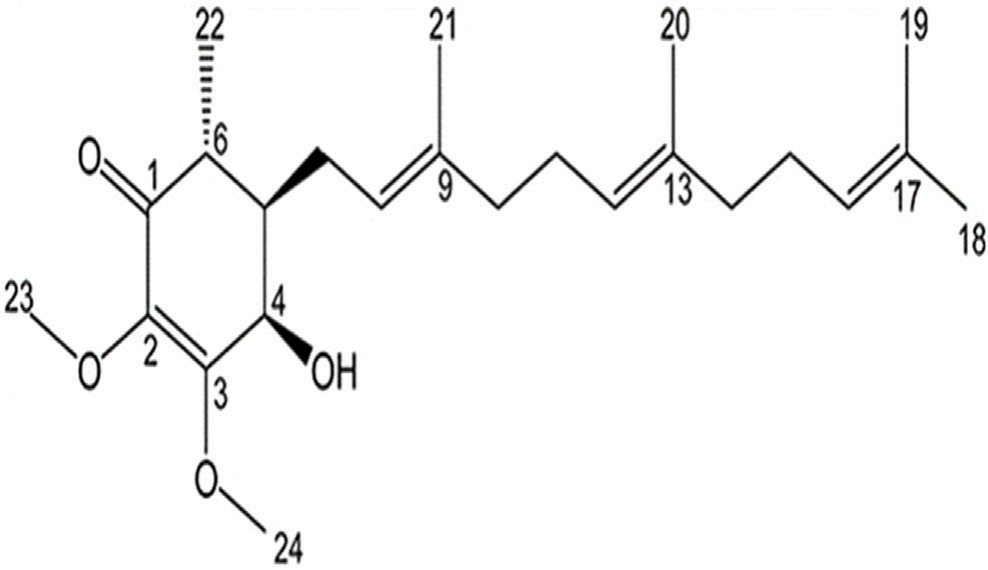
Chemical structure of antroquinonol.

Participants in the test group consumed Golden**-**Antrodia camphorata, while those in the placebo group were orally administrated with tablets without Golden-Antrodia camphorata. Both tablets were indistinguishable in appearance or taste. The components of the Golden-Antrodia *camphorata* tablets are listed in Table 1. Antroquinonol is the main bioactive ingredient in Golden-*Antrodia camphorata* tablets. Figure 1 illustrates the chemical structure of antroquinonol. The molecular formula of antroquinonol is C_24_H_38_O_4_ and its molecular weight is 390.6.

**TABLE 1:** Compounds and combination percentages per tablet (600 mg).

Material name	Combination Percentage (%)	Content (mg)
Golden-*Antrodia camphorata*	50.0	300.0
Crystalline cellulose	40.7	244.2
Lactose	5.0	30.0
Sucrose fatty acid ester	2.0	12.0
Silicon dioxide	2.0	12.0
Hydroxypropylmethylcellulose	0.3	1.8
Total	100	600

### Design, Intervention, and Study Protocol

**FIGURE 2:**
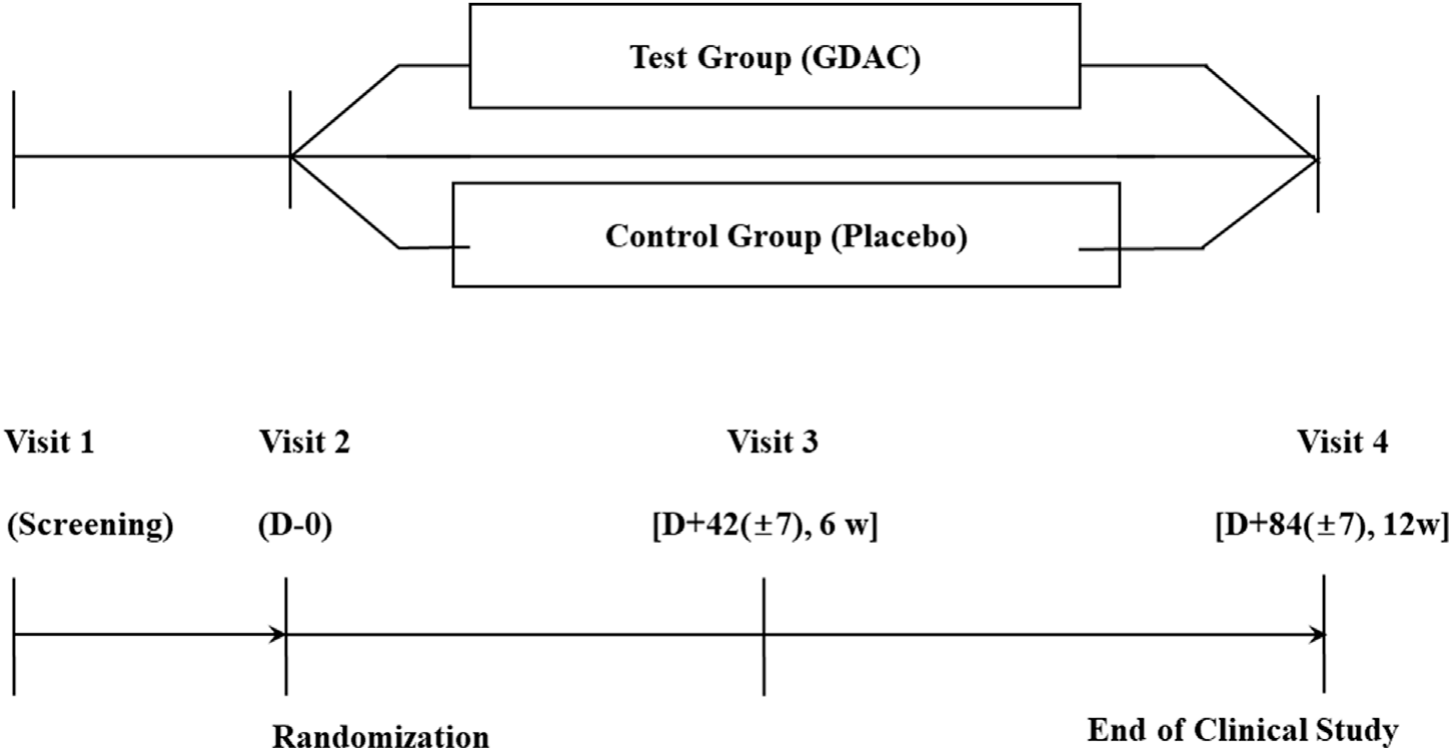
A flow diagram of the enrolment process in the human study. A subject who wishes to be enrolled in this clinical study should be given an explanation of the study and evaluated at the 1^st^ visit (Screening visit). Upon completion of our first visit, we planned to conduct a second visit within 14 days and conduct an evaluation. Three days after the second visit, a third visit was to happen, and evaluations were to take place. The fourth evaluation was conducted 84 (±7) days after the second one.

In the current multicenter randomized, double blind and placebo-controlled study, after the participants signed the consent for voluntary participation in the clinical study, their demographic features, medical and medication history, physical test results, vital signs, possibility of pregnancy (applicable only for females in their childbearing age) and clinical laboratory test results, electrocardiogram and abdominal ultrasonography findings and alcohol drinking habits were recorded before their randomization by inclusion/exclusion criteria. Participants in the test or placebo groups were treated with tablets with or without Golden-Antrodia *camphorata*, respectively, for a total of 12 weeks. The protocol of the trial specifies that safety checkpoints are held every 6 weeks, while efficacy checkpoints and safety inspections are held at the end of 12 weeks. Participants were randomly assigned to the test or placebo group at a ratio of 1:1 (Figure 2).

### Measurement of Dietary Intake

To record the dietary habits of participants during the study period, a food intake questionnaire was provided to each participant during visits 2 and 3 (Figure 2). The subject was asked to record their regular meals, as well as other meals, for 3 days in the week prior to their next scheduled visit (including 1 day on the weekend if possible). At visit days 3 and 4, the investigator evaluated the answers of the food intake questionnaire.

### Safety Investigation

Golden**-**Antrodia *camphorata* administration was evaluated based on the classification (Table 7), frequency, and severity (Table 8) of adverse events (ΑΕs), which were recorded in each participant’s AE report, and abnormal findings in clinical laboratory tests, including blood tests (Table 9), biochemical and urine tests (Table 10), vital signs (blood pressure and pulse), weight, and electrocardiogram findings. Analyzing statistically the abnormal laboratory test results recorded within each participant’s AE report, we evaluated their clinical significance.

### Statistical Analysis

Statistical analysis was carried out with SAS^®^ software (version 9.4; SAS Institute, Cary, North Carolina, United States)., A two-tailed test was performed. In all analyses, *p*-values were rounded off up to 4 decimal places. *p* < 0.05 was considered to indicate a statistically significant difference. The significance level for the efficacy, safety and demographic and nutrition analysis data was set at *p* < 0.05.

## Results

### Participant Characteristics

Herein, 188 individuals were screened to select the suitable candidates. A total of 84 subjects were excluded from the study, while the remaining 104 participants were randomly allocated to the test (*n* = 53) and placebo (*n* = 51) groups. Among them, 17 subjects were excluded due to violation of inclusion/exclusion criteria (*n* = 7), consent withdrawal (*n* = 7), follow-up failure (*n* = 1), principal investigator directed termination (*n* = 1) and prohibited combination of drug intake (*n* = 1). Finally, 80 subjects (37 in the test and 43 in the placebo groups) completed the clinical study. In all, 104 participants drank. The general characteristics of the study population and alcohol drinking habit are listed in Table 2 and 3. No statistically significant differences were observed in sex, age and high fat food intake between the two groups.

**TABLE 2:** Demographic characteristics of participants prior to treatment (PP Set).

		Test	Placebo	Total	P-value
		*n* = 37	*n* = 43	*n* = 80	
Sex [n (%)]	Male	34 (91.89)	35 (81.40)	69 (86.25)	0.1741^b^
	Female	3 (8.11)	8 (18.60)	11 (13.75)	
Age (years)	Mean ± SD	42.76 ± 12.79	43.56 ± 12.69	43.19 ± 12.66	0.7798^a^
	Range (min.-max.)	21.00-65.00	22.00-72.00	21.00-72.00	
High-fat diet^d^ [n (%)]	No	1 (2.70)	1 (2.33)	2 (2.50)	0.7287^c^
	1-2 times/week	23 (62.16)	31 (72.09)	54 (67.50)	
	3 times/week	13 (35.14)	11 (25.58)	24 (30.00)	

a P-value from two sample *t*-test.

b P-value from Chi-square test.

c P-value from Fisher’s exact test.

d High-fat diet: pork belly, fried food, pizza, chicken, hamburger, cake and jajangmyeon.

**TABLE 3:** Questionnaire on alcohol drinking habit (PP Set).

		Test	Placebo	p-value
		N = 37	N = 43	
Alcohol drinking habit (V1)	No, n (%)	0 (0.00)	0 (0.00)	-
	Yes, I enjoy alcohol drinking, n (%)	37 (100.00)	43 (100.00)	
		About (___) unit^c^/week in alcohol drinking subjects
	Mean ± SD	29.46 ± 15.42	29.12 ± 15.69	0.8610^a^
	Min, Max	8.00, 90.00	8.00, 84.00	
Alcohol drinking habit (V3)	No, n (%)	0 (0.00)	3 (6.98)	0.2448^b^
	Yes, I enjoy alcohol drinking, n (%)	37 (100.00)	40 (93.02)	
		About (___) unit^c^/week in alcohol drinking subjects
	Mean ± SD	29.78 ± 17.95	25.68 ± 16.01	0.2445^a^
	Min, Max	6.00, 90.00	4.00, 84.00	
Alcohol drinking habit (V4)	No, n (%)	1 (2.70)	3 (6.98)	0.6197^b^
	Yes, I enjoy alcohol drinking, n (%)	36 (97.30)	40 (93.02)	
		About (___) unit^c^/week in alcohol drinking subjects
	Mean ± SD	29.92 ± 17.96	25.38 ± 16.24	0.1754^a^
	Min, Max	3.00, 90.00	4.00, 84.00	

a Compared between groups; p-value for Wilcoxon rank sum test.

b Compared between groups; p-value for Fisher’s exact test.

c 2 unit = about 1/3 bottle of Soju (127 ml), 1.5 can of beer (568 ml), about 1/2 bottle of Makgeoli (425 ml), about 1/3 bottle of wine (212 ml), about 2 glasses of whisky (63.5 ml).

### Efficacy Evaluation

Tables 4 and 5 demonstrate the liver function markers and Table 6 shows the lipid levels prior to and 12 weeks after the study began. At 12 weeks following Golden**-**Antrodia c*amphorata* intake, the levels of ALT, also known as glutamate pyruvate transaminase (GPT) were reduced from the baseline levels by 11.78 ± 21.38 IU/L in the test group (*p* = 0.0002) and by 1.53 ± 19.61 IU/L in the placebo group (*p* = 0.5110), resulting in a statistically significant difference between the two groups (*p* = 0.0230). In addition, although AST/glutamic oxaloacetic transaminase (GOT) levels were decreased by 5.11 ± 10.97 IU/L in the test group (*p* = 0.0075) and increased by 1.47 ± 26.10 IU/L in the placebo group (*p* = 0.1585) at 6 weeks following tablet administration, there was no statistically significant difference identified between the groups. However, at 12 weeks after first administration, AST levels were notably reduced by 6.78 ± 8.26 IU/L in the test group (*p* < 0.0001) and 1.16 ± 18.96 IU/L in the placebo group (*p* = 0.1655) compared with the baseline values. Therefore, a statistically significant difference was observed in AST levels between the test and placebo groups (*p* = 0.0365). In addition, ALT levels were significantly reduced in the test food group at 12 weeks after ingestion (*p* < 0.05) compared to the placebo food group in specifically selected 31–51 IU/L ALT. With regards to the changes from baseline (PP set) triglyceride levels, triglyceride content was notably decreased by 36.83 ± 161.27 mg/dl in the test group (*p* = 0.0158) and elevated by 0.26 ± 86.30 mg/dl in the placebo group (*p* = 0.6696) at 12 weeks after the first intake, and a statistically significant difference was identified between the two groups (*p* = 0.0251). In males, no statistically significant differences were observed in γ-GTP levels across the groups.

**TABLE 4:** An analysis of levels of AST and ALT as well as the ratio of AST/ALT by visit (PP set).

Parameters	Treatment	Test		Placebo		P-value
		No.	Mean ± SD	No.	Mean ± SD	
AST (GOT, IU/L)	Baseline	37	32.70 ± 11.84	43	32.63 ± 15.37	0.3982^b^
	6 weeks	37	27.59 ± 8.07	43	34.09 ± 25.06	
	Change from baseline	37	-5.11 ± 10.97	43	1.47 ± 26.10	0.1839^b^
	P-value		0.0075^c^		0.1585^d^	
	12 weeks	37	25.92 ± 7.98	43	31.47 ± 20.49	
	Change from baseline	37	-6.78 ± 8.26	43	-1.16 ± 18.96	0.0365^b^
	*p*-value		<0.0001^c^		0.1655^d^	
ALT (GPT, IU/L)	Baseline	37	44.95 ± 29.34	43	40.95 ± 25.95	0.5366^b^
	6 weeks	37	35.97 ± 20.76	43	38.65 ± 22.83	
	Change from baseline	37	-8.97 ± 17.98	43	-2.30 ± 15.41	0.2329^b^
	P-value		0.0044^c^		0.0865^d^	
	12 weeks	37	33.16 ± 17.75	43	39.42 ± 28.23	
	Change from baseline	37	-11.78 ± 21.38	43	-1.53 ± 19.61	0.0230^b^
	P-value^d^		0.0002		0.5110	
AST/ALT ratio	Baseline	37	0.85 ± 0.27	43	0.90 ± 0.31	0.5856^b^
	6 weeks	37	0.91 ± 0.33	43	0.95 ± 0.37	
	Change from baseline	37	0.06 ± 0.22	43	0.05 ± 0.33	0.8169^b^
	P-value^d^		0.3079		0.3649	
	12 weeks	37	0.89 ± 0.32	43	0.90 ± 0.30	
	Change from baseline	37	0.04 ± 0.27	43	0.00 ± 0.24	0.4553^a^
	P-value^c^		0.3601		0.9663	

AST, aspartate aminotransferase; GOT, glutamic oxaloacetic transaminase; ALT, alanine aminotransferase; GTP, glutamate pyruvate transaminase.

a compared with the placebo group (two sample *t*-test).

b compared with the placebo group (Wilcoxon rank sum test).

c comparisons within groups (paired *t*-test).

d comparisons within groups (Wilcoxon signed rank test).

**TABLE 5:** The variances of AST and ALT levels in subgroups (ALT between 31 and 51 IU/L and gamma-GTP between 51 and 100 IU/L) by visit (PP Set).

Parameters	Treatment	Test	Mean ± SD	Placebo	Mean ± SD	*p*-value
		No.		No.		
AST (GOT, IU/L)	Baseline (visit 1)	6	33.50 ± 4.55	11	29.27 ± 11.73	
	6 weeks (visit 3)	6	29.33 ± 4.72	11	43.09 ± 44.69	
	Change from baseline	6	-4.17 ± 8.50	11	13.82 ± 47.44	0.552
	p-value		0.2834		0.9414	
	12 weeks (visit 4)	6	24.00 ± 2.53	11	28.27 ± 7.17	
	Change from baseline	6	-9.50 ± 5.72	11	-1.00 ± 9.22	0.0511
	p-value		0.0096		0.7265	
ALT (GPT, IU/L)	Baseline (visit 1)	6	40.50 ± 6.47	11	38.09 ± 6.70	
	6 weeks (visit 3)	6	34.33 ± 12.60	11	41.00 ± 19.03	
	Change from baseline	6	-6.17 ± 13.03	11	2.91 ± 20.95	0.4982
	p-value		0.2987		0.655	
	12 weeks (visit 4)	6	25.67 ± 6.47	11	44.82 ± 17.28	
	Change from baseline	6	-14.83 ± 5.04	11	6.73 ± 15.75	0.0088
	p-value		0.0313		0.3564	

**TABLE 6:** Levels of total cholesterol, HDL, LDL, and triglycerides based on visits (PP Set).

Parameters	Treatment	Test		Placebo		*p*-value
		No.	Mean ± SD	No.	Mean ± SD	
Total cholesterol (mg/dl)	Baseline	37	197.30 ± 41.29	43	189.65 ± 39.34	0.3995^a^
	6 weeks	37	189.05 ± 37.49	43	188.40 ± 35.82	
	Change from baseline	37	-8.24 ± 28.27	43	-1.26 ± 23.24	0.1765^b^
	*p*-value		0.0929^d^		0.7248^c^	
	12 weeks	37	196.57 ± 44.39	43	186.88 ± 34.66	
	Change from baseline	37	-0.73 ± 30.29	43	-2.77 ± 22.25	0.7303^a^
	*p*-value^c^		0.8843		0.4194	
HDL (mg/dl)	Baseline	37	53.43 ± 15.12	43	56.02 ± 13.20	0.1843^b^
	6 weeks	37	51.76 ± 12.14	43	55.35 ± 14.05	
	Change from baseline	37	-1.68 ± 9.04	43	-0.67 ± 7.22	0.5836^a^
	*p*-value^c^		0.2672		0.5435	
	12 weeks	37	53.62 ± 12.96	43	56.02 ± 12.52	
	Change from baseline	37	0.19 ± 8.88	43	0.00 ± 9.34	0.9266^a^
	*p*-value^c^		0.8976		1.0000	
LDL (mg/dl)	Baseline	37	114.95 ± 37.88	43	113.40 ± 36.26	0.8523^a^
	6 weeks	37	112.86 ± 32.86	43	110.79 ± 32.27	
	Change from baseline	37	-2.08 ± 23.37	43	-2.60 ± 24.47	0.9227^a^
	*p*-value^c^		0.5914		0.4891	
	12 weeks	37	116.30 ± 32.40	43	110.30 ± 33.23	
	Change from baseline	37	1.35 ± 27.77	43	-3.09 ± 22.61	0.4325^a^
	*p*-value^c^		0.7689		0.3748	
Triglyceride (mg/dl)	Baseline	37	219.43 ± 132.92	43	173.28 ± 99.89	0.1060^b^
	6 weeks	37	192.59 ± 134.18	43	180.09 ± 103.37	
	Change from baseline	37	-26.84 ± 165.13	43	6.81 ± 98.78	0.0667^b^
	*p*-value^d^		0.1090		0.4354	
	12 weeks	36^e^	176.56 ± 130.69	43	173.53 ± 88.06	
	Change from baseline	36^e^	-36.83 ± 161.27	43	0.26 ± 86.30	0.0251^b^
	*p*-value^d^		0.0158		0.6696	

HDL, high-density lipoprotein; LDL, low-density lipoprotein.

a compared with the placebo group (two sample *t*-test).

b compared with the placebo group (Wilcoxon rank sum test).

c comparisons within groups (paired *t*-test).

d comparisons within groups (Wilcoxon signed rank test).

e excluded from analysis since a value (2,332) deviated abnormally from the distribution of the test group (02-R044).

### Analysis of Adverse Events

The classification and severity level of adverse events and their association with Golden- Antrodia *camphorata* treatment are listed in Tables 7, 8. The survey on the severity of adverse events revealed 7 and 5 cases of mild and moderate adverse events, respectively, in the test group. In the placebo group, 3 cases of mild adverse events were recorded. No severe adverse reactions were reported in the Golden**-**Antrodia c*amphorata* treatment group. Furthermore, clinical indicators, such as heart function, blood pressure and pulse, and hematological parameters, including white blood cell, platelet and red blood cell counts, total bilirubin, albumin, blood urea nitrogen, creatinine, uric acid, total protein and glucose levels, and hematocrit and pH were also determined. However, no statistically significant changes were observed before and after treatment in both groups, since all clinical parameters were within normal ranges (data not shown). Incidence of any AEs was slightly higher in the Golden-Antrodia camphorata compared with placebo group, but the difference was non-significant. No serious AEs occurred in this clinical study.

**TABLE 7:** Adverse events associated with Golden-*Antrodia Camphorata* consumption (Safety Set).

System organ class	Test (N = 53)		Placebo (N = 51)		Total (N = 104)	
Preferred term	N (%)	Case number	N (%)	Case number	N (%)	Case number
Gastrointestinal disorders	2 (3.77)	4	0 (0.00)	0	2 (1.92)	4
Abdominal pain	1 (1.89)	2	0 (0.00)	0	1 (0.96)	2
Diarrhea	2 (3.77)	2	0 (0.00)	0	2 (1.92)	2
General disorders and administration site conditions	1 (1.89)	1	0 (0.00)	0	1 (0.96)	1
Fatigue	1 (1.89)	1	0 (0.00)	0	1 (0.96)	1
Infections and infestations	1 (1.89)	1	1 (1.96)	1	2 (1.92)	2
Bronchitis	1 (1.89)	1	0 (0.00)	0	1 (0.96)	1
Nasopharyngitis	0 (0.00)	0	1 (1.96)	1	1 (0.96)	1
Injury, poisoning and procedural complications	1 (1.89)	1	0 (0.00)	0	1 (0.96)	1
Lip injury	1 (1.89)	1	0 (0.00)	0	1 (0.96)	1
Musculoskeletal and connective tissue disorders	0 (0.00)	0	1 (1.96)	1	1 (0.96)	1
Back pain	0 (0.00)	0	1 (1.96)	1	1 (0.96)	1
Nervous system disorders	2 (3.77)	2	0 (0.00)	0	2 (1.92)	2
Headache	2 (3.77)	2	0 (0.00)	0	2 (1.92)	2
Respiratory, thoracic and mediastinal disorders	1 (1.89)	1	0 (0.00)	0	1 (0.96)	1
Rhinitis allergic	1 (1.89)	1	0 (0.00)	0	1 (0.96)	1
Skin and subcutaneous tissue disorders	2 (3.77)	2	1 (1.96)	1	3 (2.88)	3
Eczema	1 (1.89)	1	1 (1.96)	1.96	2	1.92
Urticaria	1 (1.89)	1	0 (0.00)	0	1 (0.96)	1
Totoal^a^	9 (16.98)	12	3 (5.88)	3	12 (11.54)	15

a Accumulated total (number of cases).

**TABLE 8:** The severity of adverse events and their association with consumption of Golden-*Antrodia Camphorata* (Safety set).

	Test (N = 53)		Placebo (N = 51)			Total (N = 104)		*p*-value^a^
	Case no.	Incidence (%)	Case no.	Incidence (%)	Case no.		Incidence (%)	
Severity of symptoms								
Mild	7	58.33	3	100.00	10		66.67	0.5055
Moderate	5	41.67	0	0.00	5		33.33	
Severe	0	0.00	0	0.00	0		0.00	
Association with Golden-Antrodia camphorate intake								
Clearly associated	3	25.00	0	0.00	3		20.00	0.7626
Considered to be associated	1	8.33	0	0.00	1		6.67	
Possible association	2	16.67	0	0.00	2		13.33	
Considered not to be associated	6	50.00	3	100.00	9		60.00	
Clearly not associated	0	0.00	0	0.00	0		0.00	
Unknown	0	0.00	0	0.00	0		0.00	

a *p*-value from Fisher’s exact test.

### Biochemical Blood Test in Safety Set

Tables 9, 10 summarizes the biochemical blood test results indicating the severity of safety set. No statistically significant differences were observed in the values of biochemical parameters between both groups at 12 weeks after the initiation of the study.

**TABLE 9:** The association between the blood parameters and Golden-*Antrodia Camphorata* consumption (Safety set).

Parameters	Treatment	Test		Placebo		p-value
		N = 53		N = 51		
		N	Mean ± SD	N	Mean ± SD	
RBC (10^6^/μL)	Baseline (Visit 2)	53	4.86 ± 0.40	51	4.79 ± 0.45	0.3888^a^
	12w (Visit 4)	43	4.77 ± 0.42	44	4.79 ± 0.44	
	Change from baseline	43	-0.06 ± 0.21	44	0.01 ± 0.20	0.0983^a^
	p-value^c^		0.0541		0.7322	
Hb (g/dl)	Baseline (Visit 2)	53	15.18 ± 1.15	51	14.99 ± 1.47	0.4748^a^
	12w (Visit 4)	43	15.00 ± 1.28	44	14.85 ± 1.66	
	Change from baseline	43	-0.18 ± 0.75	44	-0.09 ± 0.73	0.5399^a^
	p-value^c^		0.1136		0.4369	
Hct (%)	Baseline (Visit 2)	53	44.08 ± 3.12	51	43.63 ± 3.74	0.5100^a^
	12w (Visit 4)	43	43.60 ± 3.13	44	43.51 ± 3.94	
	Change from baseline	43	-0.37 ± 1.88	44	-0.13 ± 1.85	0.5493^a^
	p-value^c^		0.2014		0.6385	
WBC (10^3^/μL)	Baseline (Visit 2)	53	6.68 ± 1.53	51	6.49 ± 1.74	0.4724^b^
	12w (Visit 4)	43	6.73 ± 1.65	44	6.87 ± 1.82	
	Change from baseline	43	-0.01 ± 1.11	44	0.28 ± 1.62	0.0583^b^
	p-value^d^		0.6603		0.0325	
Platelet (10^3^/μL)	Baseline (Visit 2)	53	271.23 ± 45.86	51	264.86 ± 54.13	0.5186^a^
	12w (Visit 4)	43	267.49 ± 51.32	44	264.55 ± 59.87	
	Change from baseline	43	-3.30 ± 22.32	44	-0.77 ± 25.68	0.6255^a^
	p-value^c^		0.3375		0.8428	
Seg. Neutrophil (%)	Baseline (Visit 2)	53	53.46 ± 7.47	51	55.00 ± 7.76	0.3046^a^
	12w (Visit 4)	43	52.81 ± 8.60	44	54.29 ± 8.15	
	Change from baseline	43	-0.58 ± 8.42	44	-1.25 ± 6.89	0.7534^b^
	p-value		0.1862^d^		0.2368^c^	
Lymphocyte (%)	Baseline (Visit 2)	53	35.65 ± 6.82	51	34.68 ± 7.46	0.4907^a^
	12w (Visit 4)	43	35.61 ± 7.67	44	35.18 ± 7.63	
	Change from baseline	43	0.04 ± 6.30	44	1.23 ± 6.22	0.9121^b^
	p-value		0.5683^d^		0.1966^c^	
Monocyte (%)	Baseline (Visit 2)	53	7.24 ± 1.48	51	7.43 ± 2.01	0.9223^b^
	12w (Visit 4)	43	7.29 ± 1.65	44	7.52 ± 1.81	
	Change from baseline	43	-0.10 ± 1.49	44	-0.09 ± 1.63	0.7696^b^
	p-value		0.6539^c^		0.8814^d^	
Eosinophil (%)	Baseline (Visit 2)	53	3.03 ± 2.47	51	2.38 ± 1.39	0.3711^b^
	12w (Visit 4)	43	3.68 ± 2.41	44	2.44 ± 2.18	
	Change from baseline	43	0.65 ± 2.51	44	0.07 ± 1.90	0.0001^b^
	p-value^d^		0.0004		0.6629	
Basophil (%)	Baseline (Visit 2)	53	0.62 ± 0.29	51	0.52 ± 0.22	0.0934^b^
	12w (Visit 4)	43	0.61 ± 0.30	44	0.57 ± 0.22	
	Change from baseline	43	-0.01 ± 0.27	44	0.03 ± 0.23	0.5161^b^
	p-value		0.9631^d^		0.4086^c^	
MCV (fl)	Baseline (Visit 2)	53	90.81 ± 4.22	51	91.23 ± 4.46	0.3677^b^
	12w (Visit 4)	43	91.55 ± 4.48	44	91.00 ± 5.15	
	Change from baseline	43	0.46 ± 1.66	44	-0.48 ± 1.78	0.0359^b^
	p-value		0.0744^c^		0.0829^d^	

a Compared between groups; p-value for Two sample *t*-test.

b Compared between groups; p-value for Wilcoxon rank sum test.

c Compared within groups; p-value for Paired *t*-test.

d Compared within groups; p-value for Wilcoxon signed rank test.

**TABLE 10:** The association between the blood chemistry parameters and Golden-*Antrodia Camphorata* (Safety set).

Parameters	Treatment	Test		Placebo		p-value
		N = 53		N = 51		
		N	Mean ± SD	N	Mean ± SD	
Na (mmol/L)	Baseline (Visit 2)	53	141.68 ± 1.87	51	141.53 ± 2.01	0.7741^b^
	12w (Visit 4)	43	140.84 ± 1.99	44	140.82 ± 1.98	
	Change from baseline	43	-0.81 ± 2.07	44	-0.84 ± 1.84	0.9490^a^
	p-value^c^		0.0137		0.0041	
K (mmol/L)	Baseline (Visit 2)	53	4.31 ± 0.40	51	4.31 ± 0.42	0.6595^b^
	12w (Visit 4)	43	4.28 ± 0.40	44	4.33 ± 0.35	
	Change from baseline	43	0.00 ± 0.47	44	-0.01 ± 0.52	0.5460^b^
	p-value		1.0000^c^		0.9340^d^	
Cl (mmol/L)	Baseline (Visit 2)	53	102.45 ± 2.22	51	102.14 ± 2.46	0.6648^b^
	12w (Visit 4)	43	102.67 ± 2.10	44	102.30 ± 2.04	
	Change from baseline	43	0.30 ± 2.46	44	-0.18 ± 2.63	0.3788^a^
	p-value^c^		0.4257		0.6495	
Ca (mg/dl)	Baseline (Visit 2)	53	9.51 ± 0.43	51	9.52 ± 0.44	0.9403^a^
	12w (Visit 4)	43	9.40 ± 0.48	44	9.46 ± 0.44	
	Change from baseline	43	-0.10 ± 0.37	44	-0.09 ± 0.40	0.9137^a^
	p-value^c^		0.0875		0.1433	
CK (IU/L)	Baseline (Visit 2)	53	719.68 ± 4451.37	51	118.47 ± 49.52	0.3507^b^
	12w (Visit 4)	43	121.51 ± 81.26	44	117.64 ± 45.15	
	Change from baseline	43	10.70 ± 69.07	44	-0.89 ± 45.50	0.3438^b^
	p-value		0.2849^d^		0.8978^c^	
Protein (g/dl)	Baseline (Visit 2)	53	7.26 ± 0.36	51	7.25 ± 0.38	0.8565^a^
	12w (Visit 4)	43	7.20 ± 0.35	44	7.19 ± 0.40	
	Change from baseline	43	-0.06 ± 0.34	44	-0.06 ± 0.40	0.9679^a^
	p-value^c^		0.2738		0.3140	
Albumin (g/dl)	Baseline (Visit 2)	53	4.64 ± 0.23	51	4.66 ± 0.25	0.4125^b^
	12w (Visit 4)	43	4.60 ± 0.23	44	4.65 ± 0.23	
	Change from baseline	43	-0.04 ± 0.25	44	0.00 ± 0.26	0.4945^a^
	p-value^c^		0.2962		0.9542	
Glucose (mg/dl)	Baseline (Visit 2)	53	99.94 ± 16.11	51	107.35 ± 43.61	0.4823^b^
	12w (Visit 4)	43	103.30 ± 15.70	44	103.00 ± 20.43	
	Change from baseline	43	2.84 ± 13.43	44	3.75 ± 20.79	0.6191^b^
	p-value^d^		0.3670		0.1279	
T.Bilirubin (mg/dl)	Baseline (Visit 2)	53	0.78 ± 0.37	51	0.77 ± 0.35	1.0000^b^
	12w (Visit 4)	43	0.70 ± 0.38	44	0.74 ± 0.36	
	Change from baseline	43	-0.09 ± 0.35	44	-0.07 ± 0.29	0.4273^b^
	p-value		0.0889^c^		0.2041^d^	
ALP (IU/L)	Baseline (Visit 2)	53	151.53 ± 92.64	51	147.51 ± 85.67	0.9922^b^
	12w (Visit 4)	43	146.86 ± 84.60	44	142.32 ± 87.41	
	Change from baseline	43	-5.12 ± 28.17	44	2.34 ± 22.89	0.4942^b^
	p-value^d^		0.2605		0.9908	
Creatinine (mg/dl)	Baseline (Visit 2)	53	0.93 ± 0.16	51	0.91 ± 0.16	0.5198^a^
	12w (Visit 4)	43	0.90 ± 0.14	44	0.88 ± 0.15	
	Change from baseline	43	-0.03 ± 0.09	44	-0.04 ± 0.09	0.9399^a^
	p-value^c^		0.0204		0.0133	
BUN (mg/dl)	Baseline (Visit 2)	53	13.19 ± 3.49	51	12.50 ± 3.31	0.2982^a^
	12w (Visit 4)	43	13.29 ± 2.74	44	12.48 ± 3.63	
	Change from baseline	43	-0.21 ± 3.88	44	0.01 ± 3.62	0.7800^a^
	p-value^c^		0.7223		0.9802	
Uric acid (mg/dl)	Baseline (Visit 2)	53	6.12 ± 1.42	51	6.37 ± 1.45	0.3804^a^
	12w (Visit 4)	43	6.31 ± 1.64	44	6.25 ± 1.51	
	Change from baseline	43	-0.04 ± 0.96	44	-0.10 ± 0.71	1.0000^b^
	p-value		0.6192^d^		0.3534^c^	

a Compared between groups; p-value for Two sample *t*-test.

b Compared between groups; p-value for Wilcoxon rank sum test.

c Compared within groups; p-value for Paired *t*-test.

d Compared within groups; p-value for Wilcoxon signed rank test.

## Discussion

The liver function index of aspartate aminotransferase (AST; *p* < 0.0001), alanine aminotransferase AST; *p* < 0.0001), and triglyceride (*p* = 0.0158) were markedly reduced in the Golden**-**Antrodia camphorata treatment group compared with the placebo group per day for 12 weeks. According to a previous study, Golden-Antrodia camphorata pretreatment inhibited ethanol-induced AST, ALT, ROS, NO, MDA production, and GSH depletion via increased activation of Nrf-2 and the downstream antioxidant gene HO-1 through the MAPK pathway (Kumar et al., 2011). An animal model of hepatic injury (caused by a high-fat diet) showed that Golden**-**Antrodia *camphorata* inhibited lipid accumulation (Chen et al., 2019). By upregulating hepatic ADP-activated protein kinase and downregulating both sterol regulatory element-binding protein-1 protein expression and fatty acid synthase expression, Golden**-**Antrodia *camphorata* reduced hepatic lipids and inflammatory cytokines.

It has been reported that Antrodia *camphorata*, a medicinal mushroom species, exerts possible hepatoprotective effects against liver injury and alcohol-induced liver diseases (Kumar et al., 2011). In addition, *in vivo* and *in vitro* experiments revealed that antroquinonol, a bioactive component of Antrodia c*amphorata*, exerted significant anti-inflammatory and antioxidant activities via regulating nuclear factor erythroid 2-related factor 2 (Tsai et al., 2011). In addition, Golden**-**Antrodia *camphorata* displays antiviral, anti-inflammatory and antifibrotic activities (Jiang et al., 2021). To the best of our knowledge, the present study was the first to evaluate the safety and efficacy of Golden**-**Antrodia *camphorata*, derived from the ethanolic extract of the mycelium of Antrodia *camphorata*, in terms of a human clinical study.

GOT, also known as AST, and GPT, also known as ALT, are common enzymes associated with the metabolism of amino acids and proteins in the body. The normal AST value is between 5 and 40 units per liter. In patients with chronic renal failure due to vitamin B6 deficiency, AST and ALT levels are reported to be lower (Yasuda et al., 1995). Hepatocyte injury promotes the release of their contents, including ALT and AST, into the bloodstream. Chronic liver inflammation and alcoholic liver diseases are characterized by increased levels of both ALT and AST. Notably, a previous study demonstrated that Golden**-**Antrodia c*amphorata* treatment could reduce ALT and AST levels in bloodstream or liver tissues (Thiyagarajan et al., 2015). Herein, our baseline (PP group) test result was 32.70 ± 11.84, which was within the normal range. After 12 weeks of consuming Golden**-**Antrodia camphorata, ALT levels decreased by 11.78 × 21.38 IU/L in the test group (*p* = 0.0002), and by 1.53 × 19.61 IU/L in the placebo group (*p* = 0.5110), resulting in a statistically significant difference (*p* = 0.0230). Treatment of patients with alcoholic liver diseases with Golden-Antrodia *camphorata* for 12 weeks declined the serum ALT and AST levels. This finding was consistent with that observed in a previous study, where treatment with Golden**-**A. *Camphorata* could protect liver from alcohol-induced liver damage both *in vitro* and *in vivo* (Kumar et al., 2011). The results of the present study provided a scientific basis for the hepatoprotective effects of Antrodia *camphorata*. Data indicated that Golden**-**Antrodia camphorata exerted the hepatoprotective effects of Antrodia camphorata. To the best of our knowledge, the current study strongly suggested that Golden-Antrodia *camphorata* could be considered as a potential compound for treating alcoholic liver diseases.

Triglycerides are the main components of low-density lipoproteins (LDLs) and chylomicrons. Elevated levels of triglycerides in the blood reduces the content of high-density lipoproteins, while LDLs are increased and oxidized, eventually leading to atherosclerosis (Talayero and Sacks, 2011). Herein, Golden**-**Antrodia *camphorata* treatment reduced the levels of triglycerides. Similar results were observed in high-fat diet-fed mice (Chang et al., 2018). However, further studies are required to clarify the effects of Golden**-**Antrodia *camphorata* and antroquinonol on metabolic disorders.

Overall, we found that taking Golden-Antrodia camphorata for 12 weeks improved liver function in patients with liver disease and was safe. During a 12-weeks clinical trial, no adverse effects such as dizziness or other physical discomforts were reported. Golden-Antrodia camphorata at a dose level of 600 mg daily for 12 weeks was found to be safe both for healthy subjects and people with alcoholic liver diseases. Therefore, Golden-Antrodia camphorata may be a promising health food for people who suffer from alcoholic liver diseases. Further studies are needed to clarify the effects of Golden-Antrodia camphorata on metabolic disorders. The results may provide support for the use of Golden-Antrodia camphorata in the treatment of chronic diseases.

## Data Availability

The original contributions presented in the study are included in the article/supplementary material, further inquiries can be directed to the corresponding authors.
